# EREG is a risk factor for the prognosis of patients with cervical cancer

**DOI:** 10.3389/fmed.2023.1161835

**Published:** 2023-03-20

**Authors:** Tianye Li, Ruijing Feng, Bingxin Chen, Jianwei Zhou

**Affiliations:** ^1^Department of Gynecology, The Second Affiliated Hospital, School of Medicine, Zhejiang University, Hangzhou, China; ^2^Department of Obstetrics and Gynecology, The Central Hospital of Wuhan, Wuhan, China; ^3^Department of Gynecologic Oncology, Women's Hospital, Zhejiang University School of Medicine, Hangzhou, Zhejiang, China

**Keywords:** EREG, prognosis prediction, biomarker, proliferation, apoptosis, cervical cancer

## Abstract

**Background:**

Cervical cancer continues to threaten women's health worldwide. Identifying critical oncogenic molecules is important to drug development and prognosis prediction for patients with cervical cancer. Recent studies have demonstrated that epiregulin (EREG) is upregulated in various cancer types, which contributes to cancer progression by triggering the EGFR signaling pathway. However, the role of EREG is still unclear.

**Methods:**

In this study, we first conducted a comprehensive biological analysis to investigate the expression of EREG in cervical cancer. Then, we investigated the correlations between EREG expression level and clinicopathological features. In addition, we validated the effects of EREG expression on the proliferation and apoptosis of cervical cancer cells.

**Results:**

Based on the public database, we found that the expression of EREG was higher in advanced cervical cancer samples. Survival analysis showed that EREG was a risk factor for the prognosis of cervical cancer. *In vitro* experiments demonstrated that EREG knockdown undermined proliferation and promoted apoptosis in cancer cells.

**Conclusion:**

EREG plays a vital role in the progression of cervical cancer, which contributes to hyperactive cell proliferation and decreased cell apoptosis. It might be a valuable target for prognosis prediction and drug development for cervical cancer in the future.

## 1. Background

Cervical cancer remains a conundrum for gynecology clinicians and poses a serious threat to women's health worldwide (1). It is estimated that cervical cancer leads to 342,000 deaths, accounting for 7.7% of all deaths from malignancies in women (2). Due to human papillomavirus (HPV) vaccination and the use of cervical cancer screening, the incidence of cervical cancer in developed countries has been decreasing year by year. However, in low-income and developing countries, the incidence and mortality rates of cervical cancer are still high. The number of cervical cancer deaths in these regions accounts for more than 90% of global cervical cancer deaths (3). Although the overall survival of early stage cervical cancer is satisfactory after standardized treatment, the outcome of patients with locally advanced or metastatic cervical cancer is still poor (4). At the present stage, chemotherapy and radiotherapy cannot meet the unmet clinical needs (5, 6). Meanwhile, the development of novel targeted agents such as tyrosine kinase inhibitors (TKI), poly (ADP-ribose) polymerase inhibitors (PARPi), and immune checkpoint inhibitors has altered the standard treatment paradigm for cancer (7–10). Identifying key genes or signaling pathways in cervical cancer is important for risk stratification and drug development.

Hyperactivated epidermal growth factor receptor (EGFR) signaling has been reported in multiple cancer types, including but not limited to non-small cell lung cancer (NSCLC), breast cancer, bladder cancer, and colorectal cancer (11, 12). In addition, EGFR signaling is a key component driving the initiation and progression of cervical cancer. The coexistence of HPV infection and active EGFR signaling has been reported in multiple studies (13). The E5 protein of HPV could bind to the subunit of the protein pump ATPase, reduce EGFR degradation, and increase EGFR expression, eventually promoting the activation of the EGFR signaling pathway (14, 15). Moreover, the E6 protein of HPV also increases the expression of EGFR (16). Additionally, the alteration in the E6/E7 protein of HPV interferes with cervical cancer cell proliferation by decreasing EGFR stability at the posttranscriptional level (17). It has been identified that EGFR has seven ligands: EGF, EREG, amphiregulin (AREG), heparin-binding EGF-like growth factor (HB-EGF), epigen (EPGN), betacellulin (BTC), and transforming growth factor-α (TGF-α) (18). After binding with ligands, EGFR triggers the phosphorylation of downstream pathways, such as MAPK, PI3K-AKT, JAK-STAT, and PLCγ1-PKC pathways, mainly supporting cancer cell survival and proliferation (19).

As the ligand of EGFR, EREG is commonly upregulated in cancer types, such as non-small cell lung cancer, breast cancer, gastric cancer, head and neck cancer, ovarian cancer, colorectal cancer, brain cancer, and bladder cancer (20). The EREG–EGFR axis participates in tumor progression by regulating several biological functions, including cell survival, proliferation, migration, and angiogenesis (21). In NSCLC, increased EREG is robustly associated with aggressive tumor phenotypes and poor outcomes (22, 23). Similarly, in gastric cancer and colorectal cancer, upregulated EREG also predicts the shorter survival of patients (24, 25). Generally, EREG is an unfavorable factor for the outcomes of patients with tumors. However, there are still rare studies investigating the role of EREG in cervical cancer.

In this study, we calculated the correlations between EREG expression and clinical-pathological characteristics and prognosis of patients with cervical cancer. Moreover, we measured the effects of EREG knockdown on the proliferation and apoptosis of cervical cancer cells. Collectively, we showed that EREG might be a promising prediction biomarker and treatment target for cervical cancer in the future.

## 2. Materials and methods

### 2.1. Data available source

All expression profiles and clinicopathological parameters were obtained from The Cancer Genome Atlas (TCGA) and TCGA TARGET GTEx, a combined cohort of TCGA, TARGET, and Genotype Tissue-Expression (GTEx) databases, and downloaded from the UCSC website (https://xenabrowser.net/). The web addresses of online websites and online analysis tools are presented in the context.

### 2.2. Expression level analysis

The expression level of EREG in 44 different types of cancer was collected. The survival data were extracted from a previous follow-up study (26). Samples with a follow-up duration of < 30 days were excluded. Cancer types with < 10 cases were omitted. Log2 (x + 0.001) transformation was performed for each expression value. Coxph function of R package survival (version 3.2-7) was used to establish a Cox proportional hazards regression model, and a forest map was conducted. The correlation between expression and clinicopathological parameters was calculated and analyzed using the online tool Kaplan-Meier Plotter (http://kmplot.com/analysis/) and the GraphPad Prism software (version 8.0).

### 2.3. Functional enrichment analysis and correlation analysis

The RNA-seq data were derived from the TCGA.CESC.SampleMap HiSeqV2 dataset and downloaded from http://xena.ucsc.edu/. Genes with a correlation coefficient R > 0.3 were identified as EREG-related members. The online tool Database for Annotation, Visualization, and Integrated Discovery (DAVID) (https://david.ncifcrf.gov/) was used for enrichment analysis. The Gene Ontology (GO) and the Kyoto Encyclopedia of Genes and Genomes (KEGG) were adopted in enrichment analysis.

### 2.4. Protein interaction analysis

A protein interaction network analysis was employed to investigate EREG-associated proteins. The online tool STRING was used in protein interaction network analysis (https://cn.string-db.org/).

### 2.5. RNA methylation analysis

A uniformly normalized Pan-Cancer online dataset TCGA-TARGET-GTEx derived from the UCSC (https://xenabrowser.net/) database was downloaded. Subsequently, we extracted the EREG and 44 marker genes of three types of RNA modifications. The primary solid tumor, primary tumor, primary blood-derived cancer bone marrow, and primary blood-derived cancer peripheral blood samples were collected and analyzed, while normal samples were excluded from the analysis. Then, further log2 (x + 0.001) transformation was performed for the expression matrix. Finally, Pearson correlations for RNA methylation modification marker genes and EREG were calculated.

### 2.6. Immune-associated analysis

The correlation between EREG expression and immunoregulatory genes was investigated using the SangerBox online platform (http://sangerbox.com/tool.html). Ultimately, the Pearson correlations between EREG and five immune pathway marker genes were calculated. In addition, the TIMER online platform (http://timer.comp-genomics.org/) was adopted to explore the relationship between EREG and immune cells in cervical cancer.

### 2.7. Cell culture

SiHa (cervical squamous cancer cell line) and Caski (omentum-metastasized cervical cancer cell line) were purchased from the American Type Culture Collection (ATCC, Manassas, VA, USA) and maintained at the Second Affiliated Hospital, School of Medicine, Zhejiang University Laboratory (Hangzhou, China). SiHa cells were cultured with Dulbecco's Modified Eagle Medium (DMEM), and Caski were cultured in RPMI 1640 containing a concentration of 10% fetal bovine serum (FBS). All of the cells were cultured in the incubator with 5% CO_2_ at a temperature of 37°C.

### 2.8. RNA interference

The small interference RNA (siRNA) was structured by Guangzhou RiboBio. The interference RNA sequences were as follows: siEREG#1 (CCACCAACCTTTAAGCAAA), siEREG#2 (GCATCTATCTGGTGGACAT), and siEREG#3 (GGCTCAAGTGTCAATAACA).

### 2.9. Quantitative analysis with RT-PCR

The sample was disrupted and solubilized using Trizol (Takara Bio.). Then trichloromethane was used to extract the RNA. The aqueous phase containing total RNA was further purified by isopropanol and ethanol. The resulting product was resolved by 0.1% DEPC, and residual DNA was wiped off with a gDNA wiper (a component of HiScript III RT SuperMix). Sample mRNA was reverse-transcribed into cDNA with HiScript III RT SuperMix for qPCR (Vazyme, Nanjing, China). Then, cDNA was quantitatively analyzed by RT-PCR using an iTaqTM Universal SYBR Green Supermix (Bio-Rad, #1725125) and a 7,500 real-time PCR instrument (Applied Biosystems). The primer sequences used are as follows: GAPDH Forward Primer, 5′-TGTGGGCATCAATGGATTTGG-3′; Reverse Primer, 5′-ACACCATGTATTCCGGGTCAAT-3′; EREG Forward Primer, 5′-GTGATTCCATCATGTATCCCAGG-3′; and EREG Reverse Primer, 5′-GCCATTCATGTCAGAGCTACACT-3′.

### 2.10. Cell counting kit-8 and clone formation assay

The 96-well plate was seeded with 2,500 cervical cancer cells per well. Using the Cell Counting Kit-8, the optical density value (OD value) at 450 nm wavelength, reflecting the vitality of the cells, was discovered after being treated for 48 h (DOJINDO). Utilizing GraphPad Prism 8, data analysis for the cell viability experiments was carried out (San Diego, CA). Data were fitted using the four-parameter logistic equation to derive the log (concentration)-response curves (for IC50 values). In a 12-well plate, 1,000 cells, after transfected with siRNA for 24 h, were put into each well. The clonal cell aggregation was given medication or a new medium after being grown for 48 h. siRNA was transfected into the cell aggregation at 7 days again for guaranteeing the effect of RNA interference. The cultured plate was then collected after 7 days, and the clones were dyed with crystal violet. The stained clonal cell aggregation was processed and analyzed using ImageJ software. Statistical differences were analyzed using Student's *t*-test, and a *P-*value of < 0.05 was considered significant.

### 2.11. Cell apoptosis assay

The cells were collected after being treated for 24 h. The AnnexinV-FITC/PI Apoptosis Detection Kit (BD556547) was subsequently used to dye the cells, and a flow cytometry device (Beckman) was used to measure the cell apoptosis rate. In each sample, three accessory wells were present. The apoptosis rate differences across groups were compared pairwise by an unpaired *t*-test, and a *P*-value of < 0.05 was regarded as statistically significant. The flow cytometry data were analyzed using the FlowJo V software.

### 2.12. Statistical analysis

Data in this study were all statistically processed and analyzed using GraphPad 8.0 software, and all data were presented as “mean ± standard deviation” (x ± SD) with at least three independent repeated experiments. The independent sample *t*-test method was used to compare the control group and experimental group. The chi-square test was used to compare the ratio's statistical significance. A *P*-value of < 0.05 was considered statistically significant. The Pearson correlation analysis was used to analyze the correlation between the two genes.

## 3. Results

### 3.1. The clinical significance of EREG in cervical cancer

**Figure 1:**
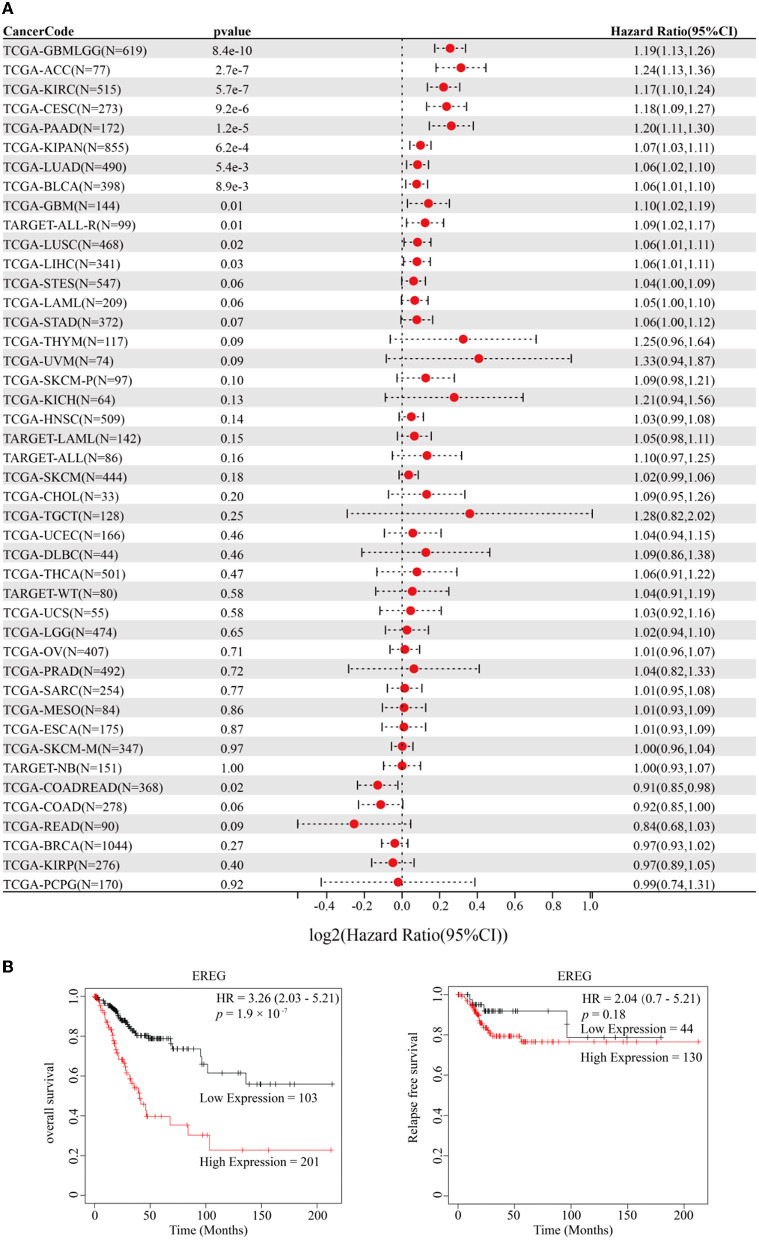
Prognosis analysis of EREG in Pan-Cancer and patients with cervical cancer. **(A)** The forest map delineated the relationship between EREG expression and overall survival in 44 types of cancer. The cancer codes and corresponding full terms are listed in Supplementary Table 1. **(B)** The overall survival and relapse-free survival of EREG in patients with cervical squamous cell carcinoma and endocervical adenocarcinoma were presented by Kaplan–Meier Plotter (http://kmplot.com/analysis/).

**Figure 2:**
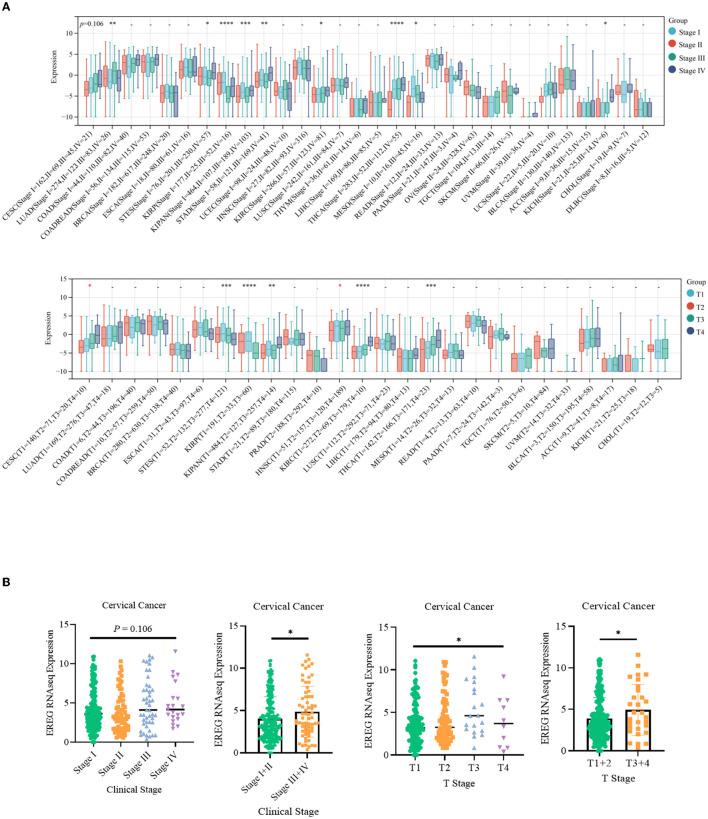
Clinical feature analysis of EREG in Pan-Cancer and patients with cervical cancer. **(A)** The relationship between EREG expression and clinical features (clinical stage and T stage) in Pan-Cancer. **(B)** The relationship between EREG expression and clinical features in cervical cancer. “*” represented *p* < 0.05, indicated statistically significant.

The standardized datasets and prognostic outcomes (overall survival) were collected. An increased level of EREG was a risk prognostic factor in the following types of cancer: glioma, adrenocortical carcinoma, kidney renal clear cell carcinoma, cervical cancer, pancreatic adenocarcinoma, Pan-kidney cohort, lung adenocarcinoma, bladder urothelial carcinoma, glioblastoma multiforme, acute lymphoblastic leukemia, lung squamous cell carcinoma, and liver hepatocellular carcinoma (Figure 1A; Supplementary Table 1). Using the Kaplan-Meier Plotter curve [Kaplan-Meier Plotter (kmplot.com)], we investigated the relationship between survival data (overall survival and relapse-free survival) and the EREG expression condition of cervical cancer (Figure 1B). The findings revealed that EREG overexpression was associated with a poor prognosis in patients with cervical cancer. Furthermore, we investigated the relationship between the EREG expression condition of patients with cervical cancer and clinical significance, including stage status and T status. The clinical stage analysis showed that EREG expression was increased in Stages 3–4 and T3–4 tumors rather than in the early stage (Figures 2A, B). It also showed that increased EREG expression resulted in worse clinical outcomes in cervical cancer.

### 3.2. Functional analysis and correlation analysis of EREG in cervical cancer

**Figure 3:**
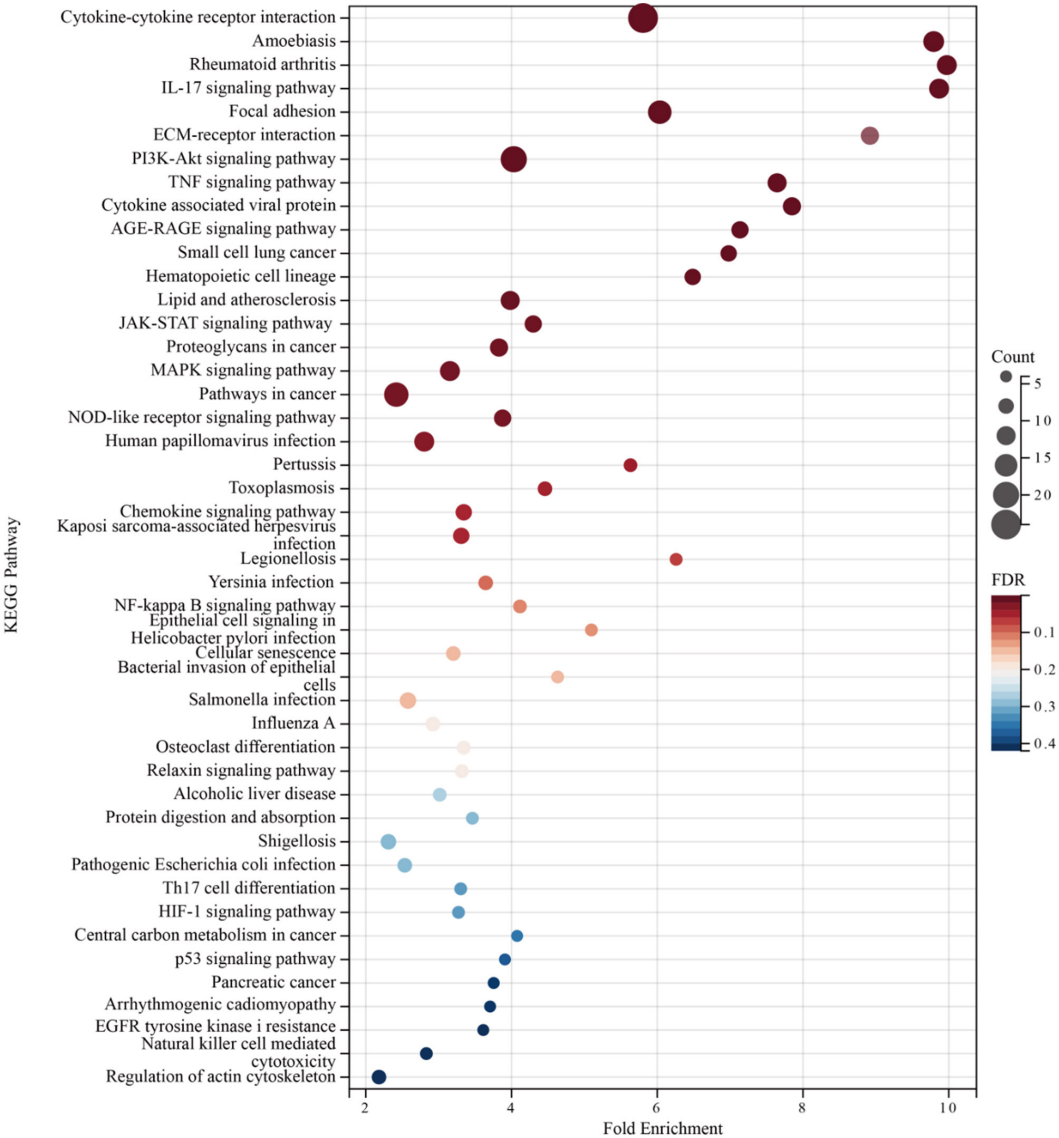
KEGG pathway enrichment analysis of EREG-correlated genes. The bubble diagram shows the enriched KEGG pathway terms for the genes correlated with EREG in the TCGA CESC dataset. The size of the bubble represents the number of associated genes included in the term. The shade of the color represents the FDR (false discovery rates).

**Figure 4:**
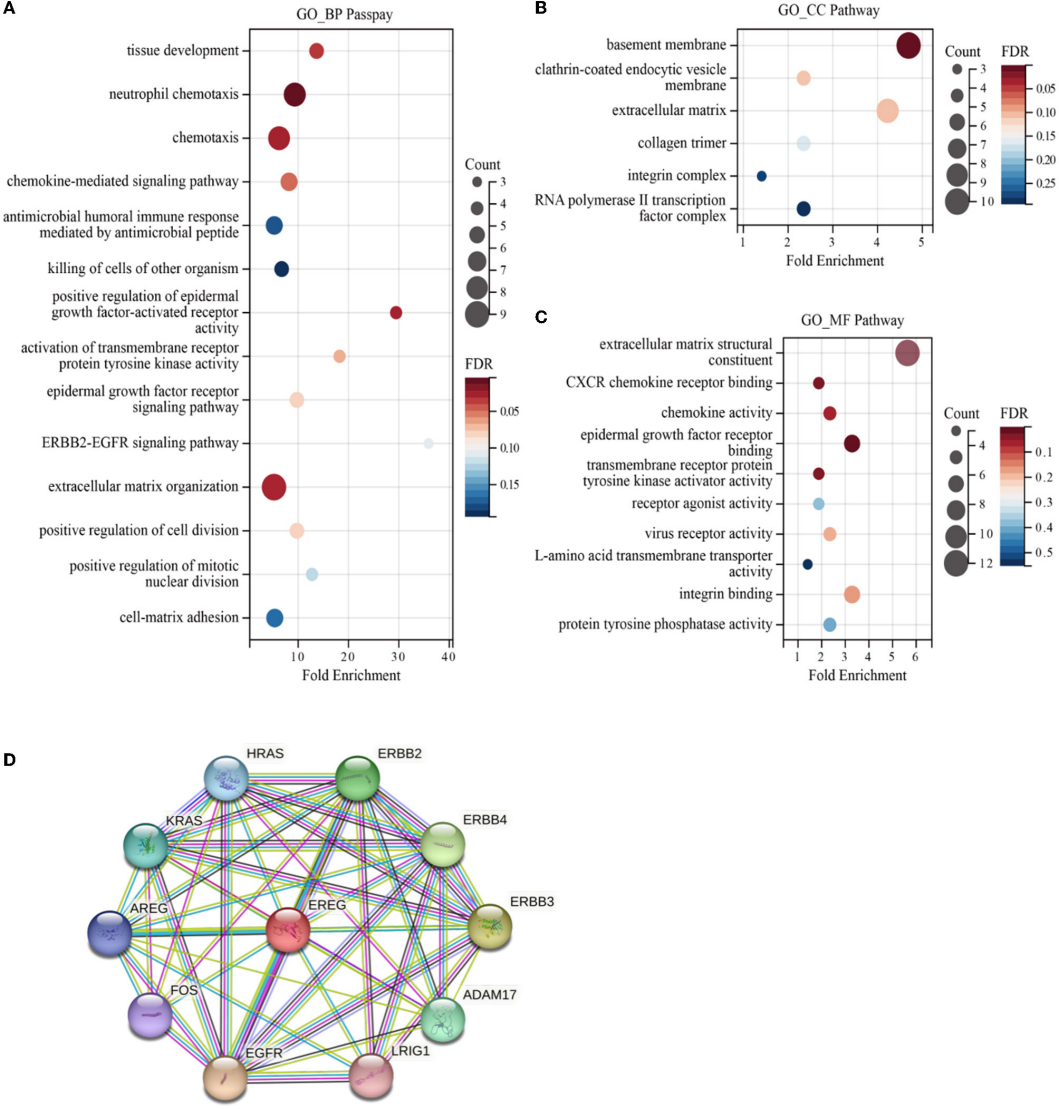
GO enrichment of EREG-correlated genes and protein interaction analysis of EREG. The bubble diagrams show the GO_BP (biological process) **(A)**, GO_CC (cellular component) **(B)**, and GO_MF (molecular function) pathway terms **(C)** of the genes correlated with EREG in the TCGA CESC dataset, respectively. The size of the bubble represents the number of associated genes included in the term. The shade of the color represents the FDR (false discovery rates). **(D)** Protein interaction network sketch map in STRING [STRING: functional protein association networks (string-db.org)]. The center of the map was EREG, and the periphery sphere represents the proteins predicted to interact with EREG.

**Figure 5:**
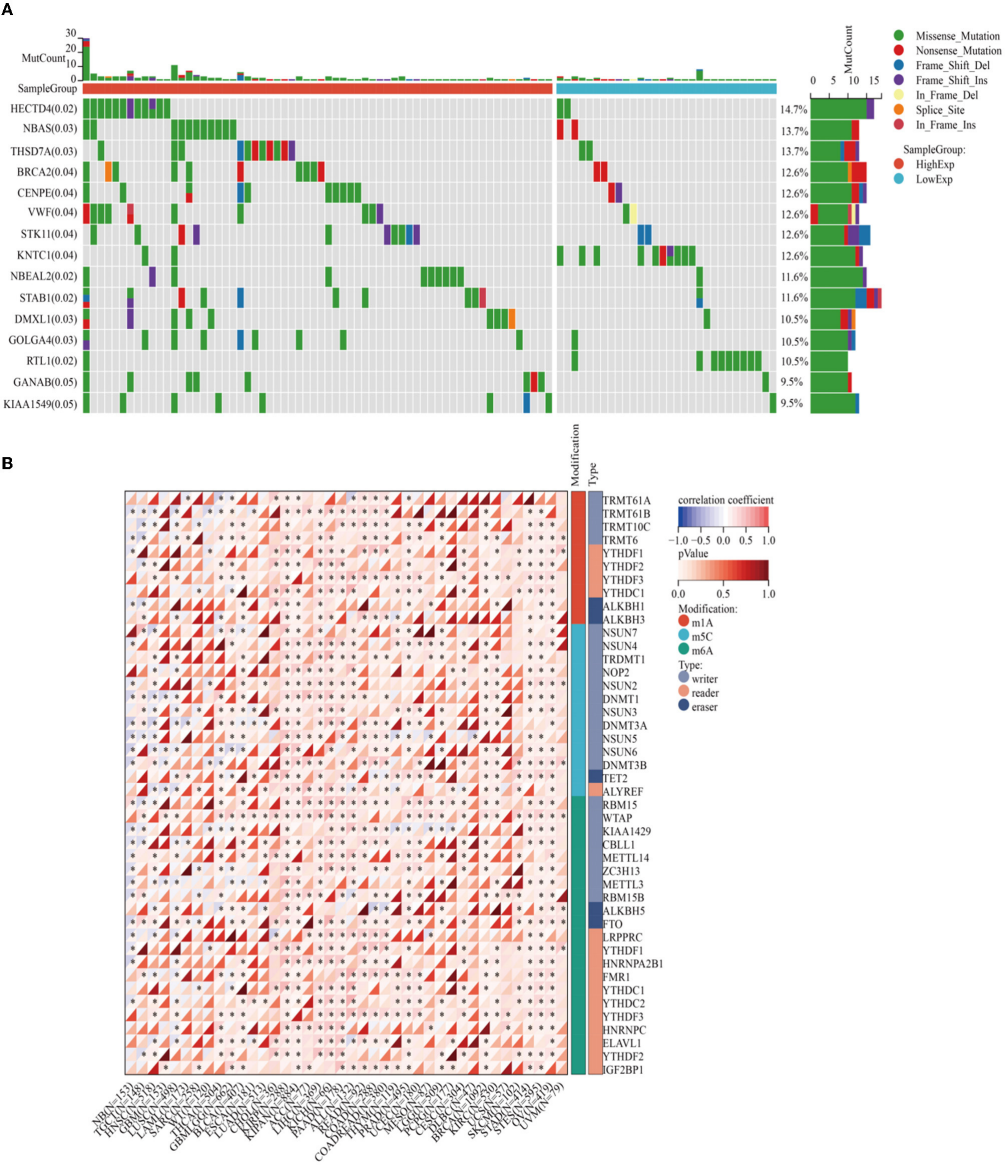
Genetic alteration analysis and RNA methylation analysis of EREG in cervical cancer. **(A)** The waterfall plot represents the genetic mutation discrepancies in a different expression of EREG in cervical cancer. The annotations in parentheses behind the gene symbol represent the *p*-value of the mutation discrepancy. **(B)** The map represents the relationship between EREG expression and the distribution of crucial RNA methylation modification genes. “*” represented *p* < 0.05, which indicated statistical significance.

Enrichment analyses of the KEGG and GO pathways were performed using the DAVID online platform. The findings suggested that EREG may play a role in a number of cancer-related molecular pathways, including those involving the EGFR biological process, the extracellular matrix structure, the interaction between cytokines and their receptors, and the PI3K-AKT, JAK-STAT, MAPK, and NK-B intracellular biological processes (Figures 3, 4A–C). Besides, protein interaction network analysis showed that the EREG could interact with or combine with the RAS family (H-Ras and K-Ras) and the ERBB family (ERBB2, ERBB3, and ERBB4), as well as its receptor EGFR (Figure 4D). In addition, genetic alteration analysis was carried out to investigate the underlying mutation-derived biological process alternatives. The findings revealed that cervical cancer tissue with a higher EREG level had higher mutative frequencies of HECTD4, NBAS, THSD7A, BRCA2, CENPE, VWF, STK11, NBEAL2, STAB1, DMXL1, GOLGA4, GANAB, and KIAA1549. Meanwhile, the cervical cancer tissue with a lower level of EREG just harbored higher mutative frequencies of KNTC1 and RTL1 (Figure 5A). Furthermore, the RNA modification analysis revealed that EREG expression was significantly correlated with the RNA methylation modification reader genes, including m1A reader (YTHDF1, YTHDF2, YTHDF3, and YTHDC1), m5C reader (ALYREF), and m6A reader (YTHDF1, YTHDF2, YTHDF3, YTHDC1, YTHDC2, and HNRNPA2B1) (Figure 5B). The aforementioned results delineate a potential biological process that, through RNA methylation regulation, EREG triggered various signaling factors dysregulation. The aforementioned factors collectively caused adverse prognostic events in cervical cancer.

### 3.3. Immune-associated analysis of EREG in cervical cancer

**Figure 6:**
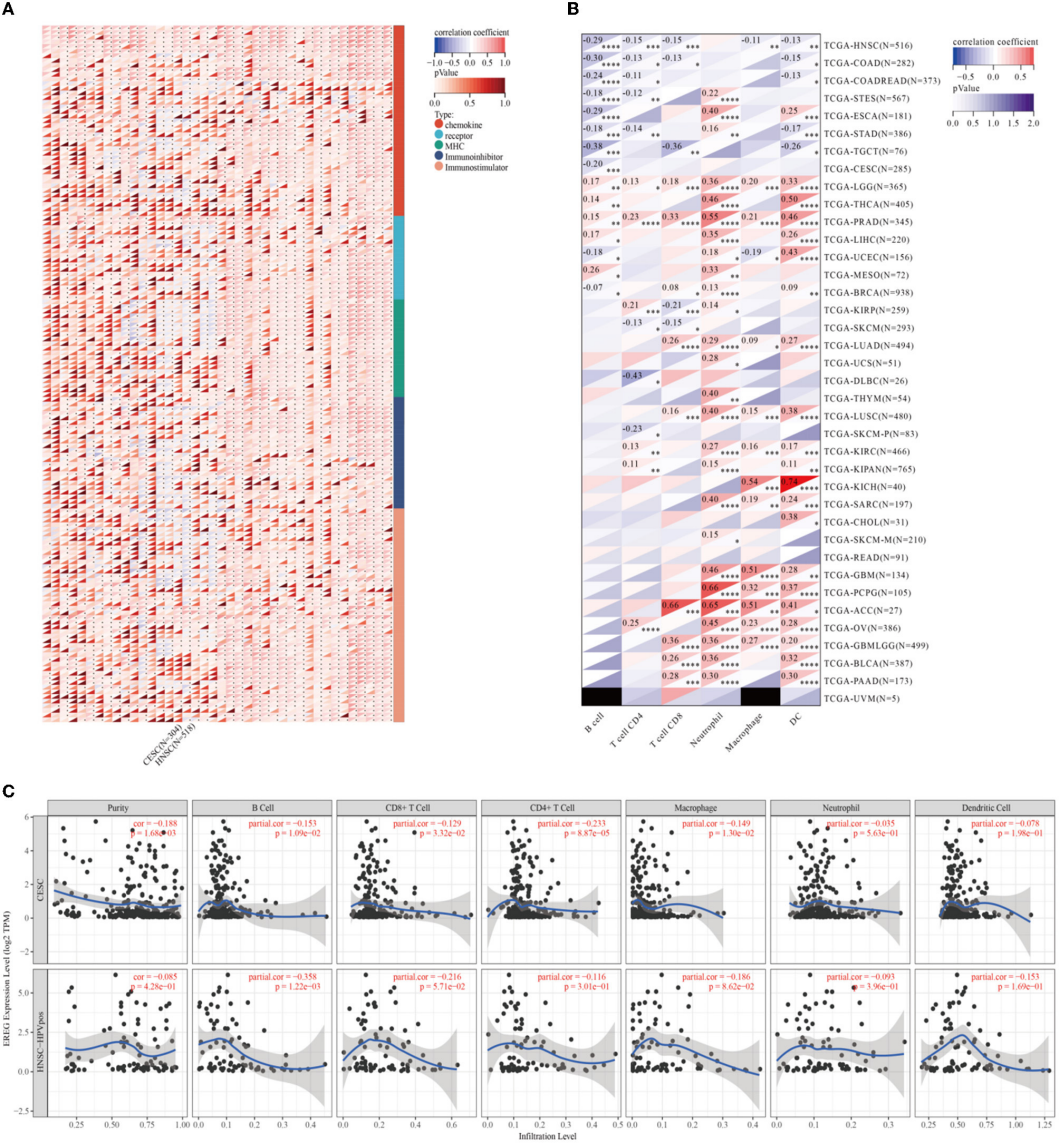
Immune-associated analysis of EREG in Pan-Cancer, cervical cancer, and HPV positive head and neck squamous cell cancer. **(A)** The map represents the relationship between EREG expression and the expression of immune regulatory genes. The types of genes were divided into chemokine, receptor, immunoinhibitory, and immunostimulator. **(B)** The correlation between immunocyte infiltration and expression of EREG in Pan-Cancer. **(C)** The correlation between immunocyte infiltration and expression of EREG in cervical cancer and HPV-positive head and neck squamous cell cancer. “*” represents *p* < 0.05, “**” represents *p* < 0.01, “***” represents *p* < 0.001, and “****” represents *p* < 0.0001.

Epiregulin was positively correlated with most chemokines, such as CXCL1, CXCL2, CXCL3, CXCL5, CXCL6, CXCL8, and CCL20, in most types of cancer, including cervical cancer. However, it was negatively correlated with some kinds of chemotactic cytokines, such as CXCL14, CCL14, CXCL17, and CX3CL1. The prognostic analysis showed that the overall survival of CXCL1, CXCL2, CXCL3, CLCL6, CXCL8, and CCL20 were all risk factors in cervical cancer, with the hazard ratios of 2.29 (*p* = 0.00072), 2.41 (*p* = 0.00016), 2.41 (*p* = 0.00021), 2.29 (*p* = 0.00034), 1.61 (*p* = 0.045), 2.97 (*p* = 1.2e-5), and 2.02 (*p* = 0.0071), respectively. In contrast, CXCL14, CCL14, CXCL17, and CX3CL1 were favorable factors, whose hazard ratios were 0.41 (*p* = 0.00017), 0.67 (*p* = 0.12), 0.61 (*p* = 0.0397), and 0.46 (*p* = 0.00095). A lot of studies have illustrated that the expression of chemokines in cervical cancer could result in different tumoral biological effects that strikingly affect the outcomes of patients (27–31). The results of the analysis showed that the expression of EREG was commonly positive relative to the adverse chemokine clusters. Besides, EREG was related to immunostimulator pathway genes rather than immunosuppressor genes (Figure 6A; Supplementary Figure 1). Furthermore, the TIMER analysis showed the relationship between EREG and immune cells. HPV-positive head and neck squamous cancer, which was considered to share the same etiology and pathology as cervical cancer, was also presented to explore the potential immune-associated mechanisms. The TIMER analysis suggested that the expression of EREG seemed negative relative to the infiltration level of most types of immune cells, including B lymphocytes, CD8+ T cells, CD4+ T cells, macrophages, neutrophil cells, and dendritic cells, both in cervical cancer and head and neck squamous cancer (Figures 6B, C). The results indicated that EREG might diminish the immune cell infiltration in the tumor microenvironment. Additionally, head and neck squamous cancer also shared the same immune regulation characteristics with cervical cancer (Figure 6A; Supplementary Figure 1), which indicated that the HPV infection might interact with EREG and together lead to cancer immune regulation dysfunction. The contradiction between the immune regulation gene analysis and immune cell infiltration analysis of EREG in cervical cancer reflected the dual character of immune regulation. However, more investigation into how EREG plays a role in the tumor immune microenvironment is needed, both *in vivo* and *in vitro*.

### 3.4. Knockdown of EREG undermined the proliferation of cervical cancer

**Figure 7:**
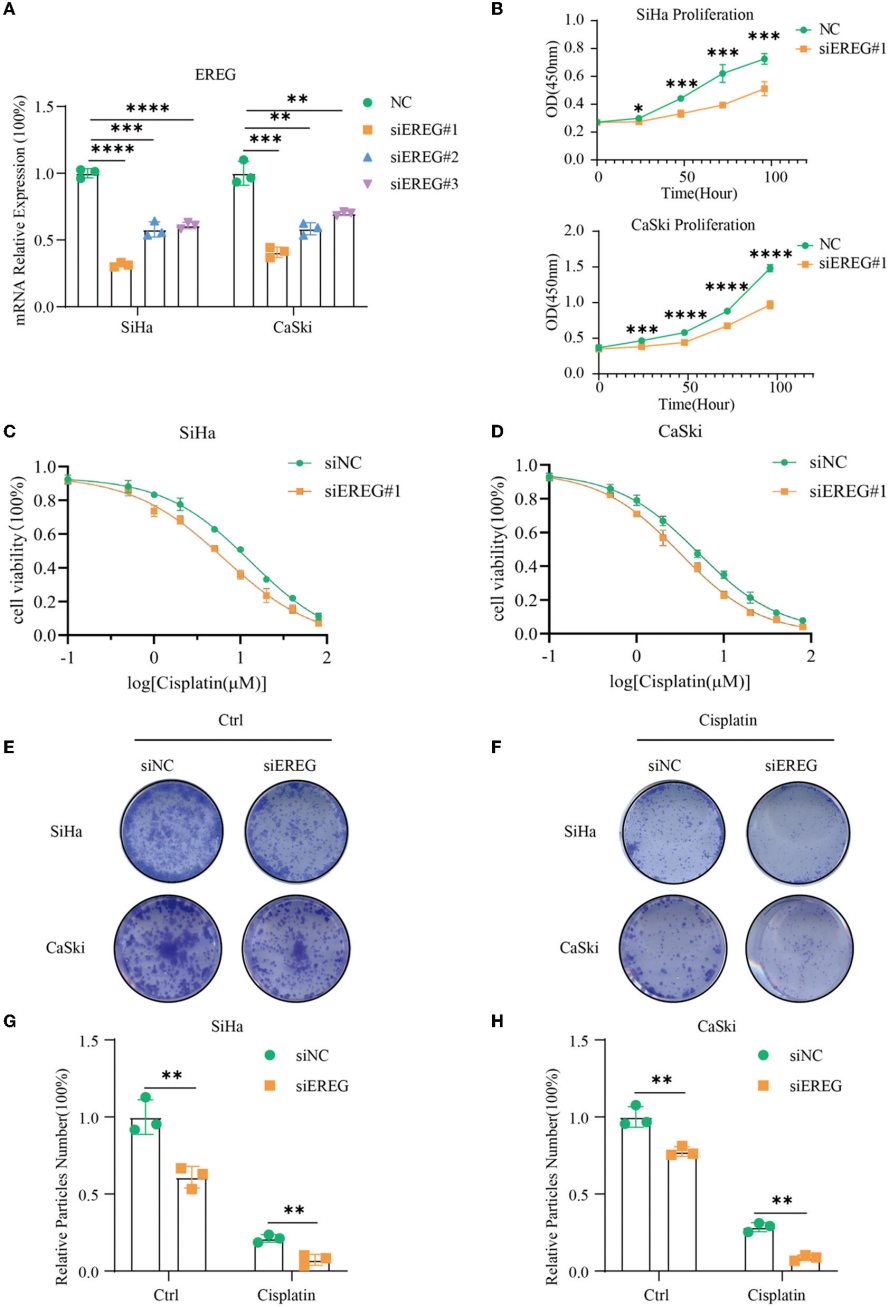
The alterations in biological behavior when EREG was knocked down in cervical cancer. **(A)** Real-time PCR was used to detect mRNA levels in SiHa and CaSki cells transfected with siEREG at 48 h. **(B)** The CCK8 cell viability curve was used to detect the proliferation of cervical cancer cells with different EREG expression levels. The CCK8 cell viability curve was used to detect the relative cell viability of SiHa **(C)** and CaSki cells **(D)** transfected with siEREG at different cisplatin concentration gradients after 48 h. **(E)** The clone formation of SiHa and CaSki transfected with siEREG. **(F)** The clone formation of SiHa and CaSki transfected with siEREG was followed by cisplatin treatment for 48 h. Histogram meant the relative clone particle numbers of SiHa **(G)** and CaSki **(H)** with or without cisplatin treatment. “*” represents *p* < 0.05, “**” represents *p* < 0.01, “***” represents *p* < 0.001, and “****” represents *p* < 0.0001.

siRNA was used to downregulate the expression of EREG in SiHa and CaSki, and the most effective siEREG#1 was selected for further experiments (Figure 7A). EGFR, which is robustly associated with EREG, is a crucial biological factor in cell proliferation. Therefore, it is apparent to detect the proliferation of cervical cancer with different EREG expression statuses. The proliferation assay using CCK8 showed that the knockdown of EREG could impede cervical cancer cell proliferation (Figure 7B). Additionally, the clone formation assay confirmed the phenomenon (Figures 7E, G, H).

### 3.5. Knockdown of EREG-induced apoptosis in cervical cancer

**Figure 8:**
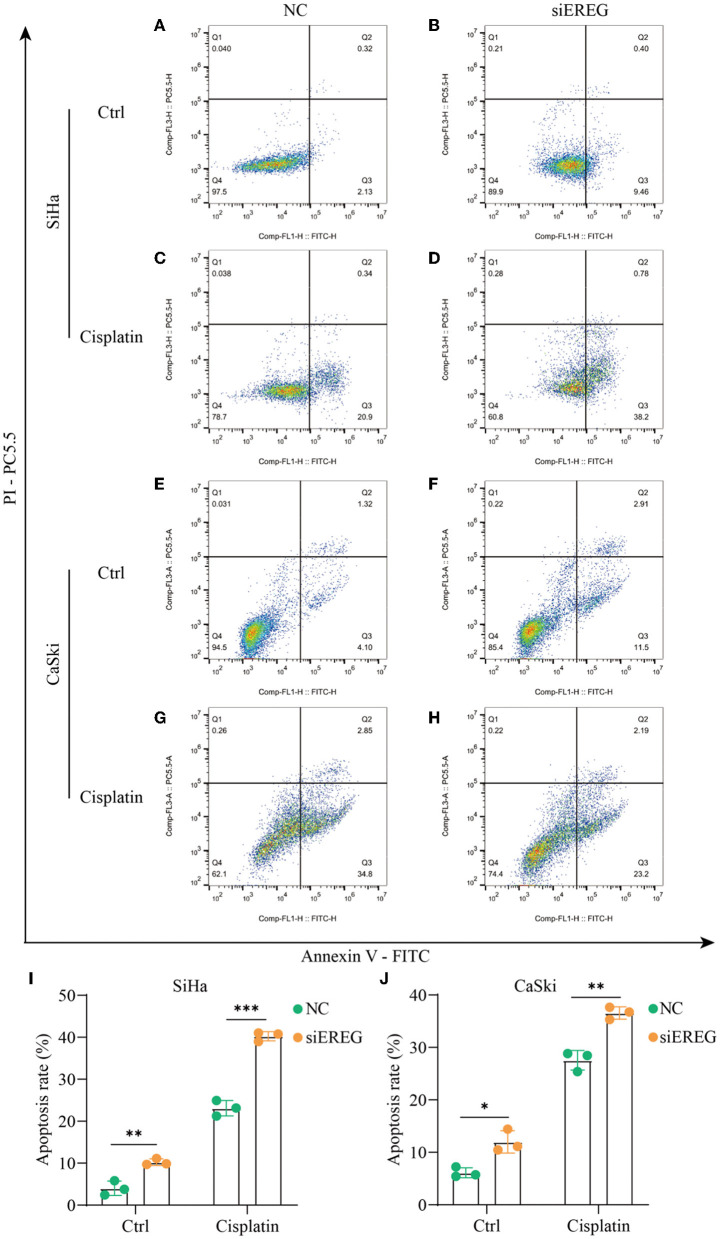
The changes in apoptosis when EREG was knocked down in cervical cancer cells with or without cisplatin treatment. Flow cytometry showed the apoptosis rate of SiHa cells after siRNA transfection, followed by treatment with 10 μM cisplatin **(C, D)** or not **(A, B)** for 48 h. Flow cytometry exhibited the apoptosis rate of CaSki cells after siRNA transfection, followed by treatment with 5 μM cisplatin **(G, H)** or not **(E, F)** for 48 h. The histogram showed the apoptosis rate of SiHa **(I)** and CaSki **(J)** with different treatments. “*” represents *p* < 0.05, “**” represents *p* < 0.01, and “***” represents *p* < 0.001.

Using the pathway enrichment analysis and protein interaction of EREG (Figures 3, 4), multiple signaling factors were identified. Therein, EREG/Ras was a potential protein interaction (Figure 4D). It was reported that the downregulation of the EREG/Ras pathway could induce cell cycle arrest and finally trigger apoptosis in hepatoma cells (32). Therefore, the apoptosis analysis was undertaken to detect the apoptosis rate of cervical cancer cells with different EREG expression levels. The results verified the putative EREG pathway and indicated the cervical cancer cells would trend to apoptosis when the EREG declined (Figures 8A, B, E, F, I, J).

### 3.6. Knockdown of EREG-sensitized cervical cancer to cisplatin

Since EREG downregulation could trigger cervical cancer apoptosis, it is logical that declining EREG could be a promising therapy for cervical cancer. However, the single gene's interference usually makes no difference because of the compensation of other signaling pathways (33). As is well known, cisplatin is the canonical chemotherapy for cervical cancer. Therefore, to further explore the effect variation of cisplatin on cervical cancer cells as the expression of EREG is downregulated, the CCK8 cell viability assay was conducted (Figures 7C, D). Both SiHa and CaSki with downregulated EREG displayed more vulnerability to cisplatin than the negative control ones through IC50 value analysis (Table 1). Meanwhile, the number of clone formation particles in cervical cancer cells with EREG knocked down was robustly less than the negative control ones following the cisplatin treatment (Figures 7F–H). Furthermore, the apoptosis rate of cervical cancer cells with EREG downregulated was significantly higher than the negative control ones in the treatment with cisplatin (Figures 8C, D, G, H–J). The results suggested that the knockdown of EREG could induce the sensibilization of cervical cancer on cisplatin and indicated a promising synergistic therapeutic regimen of cisplatin and EREG inhibitors.

**Table 1:** The four-parameter logistic equation of GraphPad Prism was used to determine the IC50 of cisplatin for each type of cervical cancer cell, taking into account their divergent EREG expression.

	**Cisplatin concentration of IC50 (95% confidence interval) (μM)**
SiHa-NC	12.9 (10.57–17.10)
SiHa-siEREG	6.395 (5.255–8.168)
CaSki-NC	5.011 (4.406–5.754)
CaSki-siEREG	3.123 (2.770–3.523)

## 4. Discussion

As a well-established protumorigenic signaling pathway, EGFR is frequently mutated in various types of cancer. EGFR drives tumorigenesis by enhancing pro-survival, antiapoptotic responses, proliferation, migration, invasion, angiogenesis, and vascular mimicry (34–36). Moreover, hyperactivated EGFR signaling leads to the upregulation of stemness markers, including Oct4, Nanog, CD44, and CXCR4. Upon binding with its ligands, such as EREG, the conformation of the tyrosine kinase domain of EGFR is altered, triggering autophosphorylation and intracellular signaling cascades. Besides acting as a cell surface receptor, EGFR could locate in the nucleus and function as a co-transcriptional activator or nuclear kinase (nuclear EGFR, also termed nEGFR). It has been validated that nEGFR promotes the expression of multiple oncogenes, such as *Cyclin D1, AurkA, c-Myc*, and *BCRP/ABCG2* (19). Additionally, nEGFR contributes to the resistance to chemotherapy, radiotherapy, and EGFR-targeting therapy (37–40). Nowadays, EGFR tyrosine kinase inhibitors, such as gefitinib and erlotinib, have been widely used in the clinic (41).

Epiregulin is the ligand of EGFR, eliciting a variety of biological functions mainly through EGFR-mediated tyrosine kinase activity. In the tumor microenvironment, autocrine and paracrine EREG activates the downstream pathways of EGFR to promote tumorigenesis (20). In a COX2-overexpressed bladder cancer model, EREG is identified as the most highly expressed EGF, supporting tumor cell proliferation (42). EREG promotes motility capability by activating MAPK and PI3K-AKT pathways in salivary adenoid cystic carcinoma cells (43). In head and neck squamous cell carcinoma, EREG enhances malignant transformation by activating the MAPK pathway and inducing C-Myc expression (44). Notably, fibroblast-derived EREG could support the growth of the colitis-associated neoplasm by activating the MAPK pathway in intestinal epithelial cells (45). In parallel, EREG/RAS dual knockdown leads to cycle arrest and retards liver cancer growth by regulating MAPK, PI3K-AKT, and Rb pathways (32). Although EREG expression has no significant relationship to clinicopathological features in gastric cancer, a high EREG level is an independent predictor of poor clinical outcomes for patients receiving curative surgery (46). However, there are rare studies estimating the role of EREG in cervical cancer.

In the present study, we found that high EREG expression was associated with the poor survival of patients with cervical cancer. In addition, EREG expression was increased in Stages T3–4 and 3–4 tumors. Enrichment analysis demonstrated that EREG was highly associated with cytokine–cytokine receptor interaction, PI3K-AKT signaling, TNF signaling, JAK-STAT signaling, MAPK signaling, and NK-κB signaling. Notably, EREG was also related to HPV infection. As mentioned earlier, HPV infection could trigger the EGFR pathway by upregulating EGFR expression. EREG, as well, plays an important role in tumoral immune regulation. It was found to be increased in myeloid cells across the progression of cancer (47). In this study, EREG was also found to take part in the negative immune regulation, mainly *via* chemokine processes and probably impeding immune cell infiltration, through which EREG eventually resulted in the adverse clinical event. Besides, *in vitro* experiments indicated that EREG knockdown limited cell proliferation and promoted cell apoptosis. Moreover, EREG knockdown relieved the resistance to cisplatin in cervical cancer cells. In conclusion, our data showed that EREG functioned as a driving factor in cervical cancer progression and contributed to chemotherapy resistance. However, the mechanistic investigation of how EREG contributed to the phenotype was limited, while EREG was considered to act through nEGFR and downstream pathways. A further mechanistic investigation was needed. In conclusion, it is logical that targeting EREG could be a potential strategy for cervical cancer treatment.

## 5. Conclusion

Collectively, high EREG expression predicts poor prognostic outcomes for patients with cervical cancer. EREG knockdown impairs proliferation and promotes apoptosis of cervical cancer cells. EREG would be a promising target for risk classification and drug development for patients with cervical cancer.

## Data availability statement

The original contributions presented in the study are included in the article/Supplementary material, further inquiries can be directed to the corresponding author.

## Author contributions

The study was conceived and designed by JZ. TL performed the most statistical analysis and experiments and wrote the manuscript. RF and BC participated in collecting literature and helped in revising the manuscript. All authors read and approved the manuscript.

## Supplementary material

Supplementary Figure 1 The map shows the relationship between EREG and immune regulatory genes. The clearer map presented the relationship between EREG expression and the expression of immune regulatory genes. The types of genes were divided into chemokine, receptor, immunoinhibitory, and immunostimulator. In addition, the official gene symbols of each type were listed right on the map.

Supplementary Table 1 Cancer codes and corresponding full terms.
