# Supplementary material for: EREG is a risk factor for the prognosis of patients with cervical cancer
Source: Front Med (Lausanne). 2023 Mar 20;10:1161835. doi: 10.3389/fmed.2023.1161835 (PMC10067667; doi:10.3389/fmed.2023.1161835)
Supplement: Supplementary Figure 1 — The map shows the relationship between EREG and immune regulatory genes. The clearer map presented the relationship between EREG expression and the expression of immune regulatory genes. The types of genes were divided into chemokine, receptor, immunoinhibitory, and immunostimulator. In addition, the official gene symbols of each type were listed right on the map. [file Table_1.DOCX]

**Table S1. The table showing the cancer codes and corresponding full terms.**

| Cancer Code | Full terms |
| --- | --- |
| TCGA-ACC | Adrenocortical carcinoma |
| TCGA-BLCA | Bladder urothelial carcinoma |
| TCGA-BRCA | Breast invasive carcinoma |
| TCGA-CESC | Cervical squamous cell carcinoma and  endocervical adenocarcinoma |
| TCGA-CHOL | Cholangiocarcinoma |
| TCGA-COAD | Colon adenocarcinoma |
| TCGA-COADREAD | Colon adenocarcinoma/Rectum adenocarcinoma esophageal carcinoma |
| TCGA-DLBC | Lymphoid neoplasm diffuse large B-cell Lymphoma |
| TCGA-ESCA | Esophageal carcinoma |
| TCGA-FPPP | FFPE pilot phase II |
| TCGA-GBM | Glioblastoma multiforme |
| TCGA-GBMLGG | Glioma |
| TCGA-HNSC | Head and neck squamous cell carcinoma |
| TCGA-KICH | Kidney chromophobe |
| TCGA-KIPAN | Pan-kidney cohort (KICH+KIRC+KIRP) |
| TCGA-KIRC | Kidney renal clear cell carcinoma |
| TCGA-KIRP | Kidney renal papillary cell carcinoma |
| TCGA-LAML | Acute myeloid leukemia |
| TCGA-LGG | Brain lower grade glioma |
| TCGA-LIHC | Liver hepatocellular carcinoma |
| TCGA-LUAD | Lung adenocarcinoma |
| TCGA-LUSC | Lung squamous cell carcinoma |
| TCGA-MESO | Mesothelioma |
| TCGA-OV | Ovarian serous cystadenocarcinoma |
| TCGA-PAAD | Pancreatic adenocarcinoma |
| TCGA-PCPG | Pheochromocytoma and paraganglioma |
| TCGA-PRAD | Prostate adenocarcinoma |
| TCGA-READ | Rectum adenocarcinoma |
| TCGA-SARC | Sarcoma |
| TCGA-STAD | Stomach adenocarcinoma |
| TCGA-SKCM | Skin cutaneous melanoma |
| TCGA-STES | Stomach and esophageal carcinoma |
| TCGA-TGCT | Testicular germ cell tumors |
| TCGA-THCA | Thyroid carcinoma |
| TCGA-THYM | Thymoma |
| TCGA-UCEC | Uterine corpus endometrial carcinoma |
| TCGA-UCS | Uterine carcinosarcoma |
| TCGA-UVM | Uveal melanoma |
| TARGET-OS | Osteosarcoma |
| TARGET-ALL | Acute lymphoblastic leukemia |
| TARGET-NB | Neuroblastoma |
| TARGET-WT | High-risk Wilms tumor |
