# Supplementary figures and images for: EREG is a risk factor for the prognosis of patients with cervical cancer
Source: Front Med (Lausanne). 2023 Mar 20;10:1161835. doi: 10.3389/fmed.2023.1161835 (PMC10067667; doi:10.3389/fmed.2023.1161835)

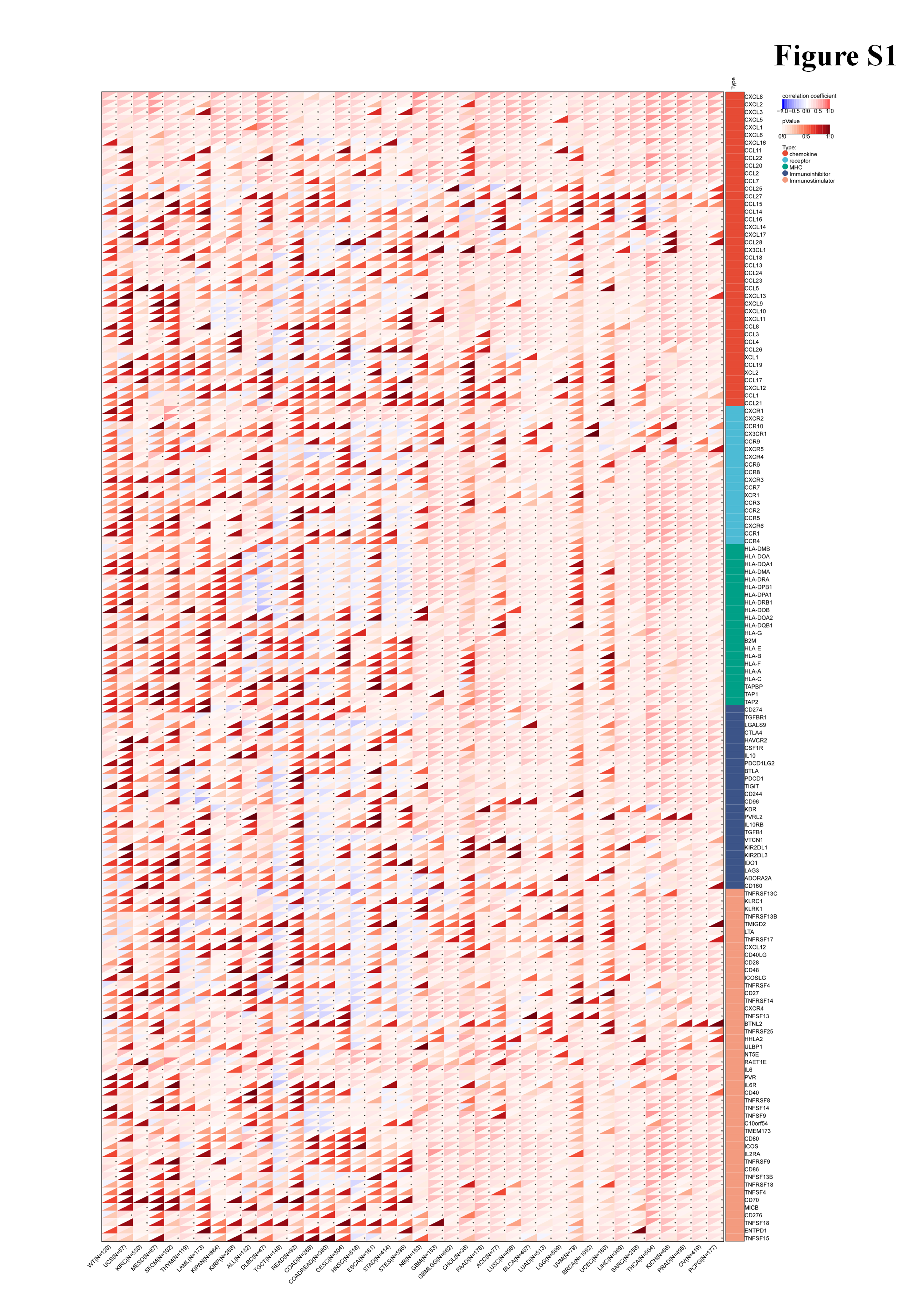

Supplement: Supplementary Table 1 — Cancer codes and corresponding full terms. [file Image_1.TIF]
